# Physical activity and depressive symptoms in adolescents: a prospective study

**DOI:** 10.1186/1741-7015-8-32

**Published:** 2010-05-28

**Authors:** Catherine Rothon, Phil Edwards, Kamaldeep Bhui, Russell M Viner, Stephanie Taylor, Stephen A Stansfeld

**Affiliations:** 1 Centre for Psychiatry, Queen Mary University of London, Barts and The London School of Medicine and Dentistry, Wolfson Institute of Preventive Medicine, Charterhouse Square, London, UK; 2 Nutrition and Public Health Intervention Research Unit, London School of Hygiene and Tropical Medicine, London, UK; 3 Institute of Child Health, University College London, London, UK; 4 Institute of Health Sciences Education, Queen Mary University of London, Barts and the London School of Medicine and Dentistry, London, UK

## Abstract

**Background:**

The frequency of mental illness amongst adolescents and its potential long-term consequences make it an important topic to research in relation to risk and protective factors. Research on the relationship between physical activity and depressive symptoms in adolescents is limited. There is a particular lack of evidence from longitudinal studies. This study examines the relationship between depression and physical activity using the Research with East London Adolescents: Community Health Survey (RELACHS).

**Methods:**

This was a prospective cohort study. Participants were recruited from three Local Education Authority boroughs in East London in 2001 from year 7 (aged 11-12) and year 9 (aged 13-14) and were followed-up in 2003. All pupils in the 28 schools that took part were eligible for the study. Of the total 3,322 pupils eligible for the survey the overall response rate was 84% (2,789 pupils). A total of 2,093 (75%) pupils were also followed-up in 2003. The sample was multiethnic (73% of respondents were non-white) and deprived. Just under half of the sample was male (49%). Depressive symptoms were measured using the Short Moods and Feelings Questionnaire (SMFQ). Logistic regression analyses were used to examine the association between physical activity and depressive symptoms both cross-sectionally and longitudinally.

**Results:**

After adjustments, there was evidence for a cross-sectional association between physical activity and depressive symptoms for both boys and girls at baseline, with a decrease in the odds of depressive symptoms of about 8% for each additional hour of exercise undertaken per week (boys: odds ratio (OR) = 0.92, 95% CI 0.85 to 0.99; girls: OR = 0.92, 95% CI 0.85 to 1.00). There was no evidence for an association between a change in physical activity from baseline to follow-up and depressive symptoms at follow-up.

**Conclusions:**

This study provides some evidence for an association between level of physical activity and decreased depressive symptoms in adolescents. Further longitudinal research of these associations is required before physical activity can be recommended as an intervention for depression in adolescents.

## Background

Psychiatric morbidity in adolescents is a concern of major public health importance. In a review of child and adolescent psychiatric disorders, the median prevalence estimate worldwide for an impairing mental health condition was 12%, although estimates varied widely [1]. The most recent and comprehensive study of children and adolescents in the UK indicated that about 10% could be defined as experiencing a clinically diagnosed mental disorder; about 4% had an emotional disorder (anxiety or depression). Those with emotional disorders were more likely to be adolescents (aged 11-16) than children (aged 5-10) and were more often girls than boys [2]. Without effective treatment, adolescents with mental health difficulties are at increased risk of academic underachievement, substance abuse, isolation and suicide [3,4]. The frequency of mental illness and its potential long-term consequences make it an important topic to research in relation to risk and protective factors. There is a growing literature from population-based cohort studies and randomised controlled trials suggesting that physical activity is associated with a reduction in depressive symptoms for adults [5]. Research on adolescents is more limited, however, with a particular lack of longitudinal studies.

No clear mechanism for the association between physical activity and depression has been established, but biochemical, physiological and psychological mechanisms have been proposed. Biochemical and physiological explanations are beyond the scope of this study and will not be discussed in detail here. There are four key psychosocial explanations for the link between physical activity and depression. The first is the distraction hypothesis. This theory posits that it is the 'time out' that physical activity provides that enhances mood rather than any biochemical or physiological mechanism. This hypothesis has been supported by experimental studies that have demonstrated that physical activity was no more efficient in improving mood than an equivalent period of relaxation [6,7]. The second explanation, the 'mastery' hypothesis, suggests that it is the completion of a task (such as learning a new sport) that brings about a sense of achievement, leading to improved mood [6,8]. The third explanation relates to the indirect effect that physical activity has on mood through providing increased opportunities for social interaction. Through participation in exercise classes or team sports, social support may become more readily available [9-11]. Finally, physical activity may relate to mood through improving self-esteem. Those that engage in programmes of physical activity have the potential to modify their body shape, leading to an improvement in self-image [12].

The role of confounding variables such as social class must also be considered. Amongst adolescents in the UK, it has been shown that those from lower socioeconomic backgrounds are more likely to exhibit psychiatric disorders [2]. Individuals of lower socioeconomic status are also more likely to live in overcrowded areas where space to exercise is less available. In areas where there is a high level of criminal activity it may be dangerous for children to play outside. The causal pathways are complex and social class is likely to influence both levels of physical activity and mental health. It may be, therefore, that any association observed between physical activity levels and depression can be explained by the confounding variable of social class.

Most studies in adolescents have been cross-sectional and have established an association between physical activity and good mental health [12-20]. There have been three major longitudinal studies. A prospective cohort study in the US found an association between reduced physical activity and increased symptoms of depression in young adolescents [21]. A study in Norway found that the number of hours spent on physical activity in a week at age 15-16 was negatively related to emotional symptoms 3 years later for boys but not for girls [10]. A longitudinal study of adolescents and young adults in Munich, Germany, found that those with regular physical activity at baseline had a lower overall incidence of mental disorder but found no association between physical activity and major depression specifically (although this may have been due to a lack of power in analyses which looked at specific outcomes) [22]. Overall therefore, the longitudinal studies that exist suggest that there is an association between physical activity and mental health but differ on how this relates to specific diagnosis and how the relationship varies by gender. A Cochrane review of exercise interventions for prevention and treatment of anxiety and depression in children and adolescents found a small effect for exercise reducing depression and anxiety in the general population but came to no firm conclusions as to its efficacy as a treatment [23].

This study examines the relationship between depression and physical activity using the Research with East London Adolescents: Community Health Survey (RELACHS) [24]. These data provide a unique opportunity to examine the association both cross-sectionally and longitudinally. The study is important for its focus on a deprived area of the UK; there is the strongest evidence for a worsening trend in adolescent mental health problems amongst socially disadvantaged populations [25].

## Methods

### Study design and setting

The data come from RELACHS, an epidemiological study of adolescents [24]. Participants were recruited from three Local Education Authority (LEA) boroughs in East London (Hackney, Newham and Tower Hamlets) in 2001. Further funding was obtained to follow-up the same participants in 2003.

### Participants

Participants in 2001 were year 7 (age 11-12) and year 9 (age 13-14) pupils from comprehensive schools in the three boroughs.

### Sampling design

**Figure 1:**
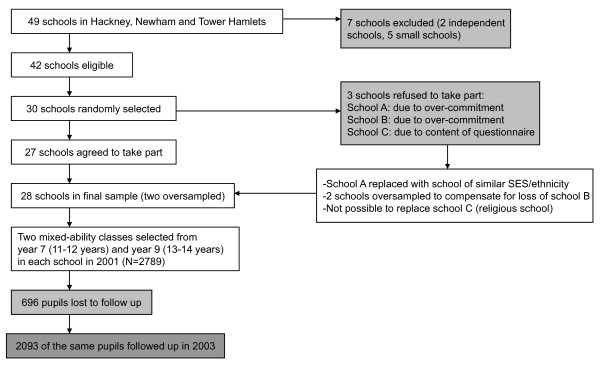
**Sampling design**.

The sample was selected using two-stage stratified random sampling (Figure 1).

### Data collection

Information about the study was given to teachers, parents and pupils a week before the school visits. Parents could choose to opt their child out. Pupils who had not been opted out were invited to take part and asked for written consent. Pupils could withdraw from the study at any time and did not have to answer questions they did not want to. A team of researchers administered the questionnaire in classrooms in one 40-50 min session. Pupils provided self-reported data on a self-completion questionnaire. Physical measurements were taken by trained researchers. Pupils were monitored to ensure they were not distressed.

### Measurement of main outcome and exposure

Depressive symptoms were measured using the Short Moods and Feelings Questionnaire (SMFQ) [26]. This includes 13 statements about emotions and behaviour over the past 2 weeks. The scores for the items were summed to produce an overall magnitude of symptoms, with a score of ≥8 indicating the presence of depression. In the original validation against the Diagnostic Interview Schedule for Children - Depressive Scale this threshold yielded a positive predictive value of 80% and a negative predictive value of 68% [27].

Questions on physical activity were taken from the Health Education Authority (HEA) survey, based on recommended levels of physical activity for this age group [28]. The variable used asked: 'OUTSIDE SCHOOL HOURS: How many hours a week do you usually exercise in your free time so much that you get out of breath and sweat?' and responses were: 'None', 'about half an hour', 'about 1 hour', 'about 2-3 hours', 'about 4-6 hours' or '7 hours or more'. For analysis this variable was recoded into a continuous variable where 'none' = 0 h, 'about half and hour' = 0.5 h, 'about 1 hour' = 1 h, 'about 2-3 hours' = 2.5 h, 'about 4-6 hours' = 5 h and '7 hours or more' = 7 h. It was not possible to use the full categorical variable, as there would have not been enough power to detect associations once the analyses were stratified by gender. Results for the full categorical variable are presented for the univariable analysis, however.

For the longitudinal analysis, a variable was created for change in physical activity between baseline and follow-up. This was calculated by subtracting the continuous physical activity variable at baseline from the continuous physical activity variable at follow-up.

### Measurement of confounders

Questions for general health were taken from the Health in Young People in England Study and questions for health in the last month were taken from HEA survey [28,29]. Questions on long-standing illness were adapted from the General Practice National Morbidity Survey [30]. Questions on smoking (frequency), drinking (frequency) and drug use (whether ever used and how recently) were taken from the Office for National Statistics surveys for teenagers [31]. Representatives from drug advice projects for young people in the area were consulted about slang names for drugs. Questions on eating were taken from the Health and Behaviours of Teenagers Study (HABITS), and were based on government guidelines of eating five portions of fruit and vegetables daily [32]. Sociodemographic measures (gender, ethnicity, religion, parental employment, eligibility for free school meals) were collected from the questionnaire and from routinely collected data. We use eligibility for free school meals as a proxy for social class. Obviously this does not capture the full range of occupational categories in the way that a measure such as the National Statistics Socio-economic Classification does. It is also a measure of income rather than occupational position. However, given that this sample is fairly homogeneous with regards to (low) social class, it is able to effectively distinguish the most deprived adolescents.

### Ethical approval

Meetings were held with a community advisory group, consisting of teachers, parents, health and social care professionals. The study protocol was approved by the East London and the City Local Research Ethics Committee.

### Data management

All data management and analysis was carried out using Stata version 10.0 (StataCorp, College Station, TX, USA). There were some missing data at baseline: 64 respondents did not have data on depression (2.3%) and 43 respondents did not have data on physical activity (1.5%). As the amount of missing data was relatively small for most of the key variables, it was considered reasonable to exclude pupils who did not have complete data.

### Statistical analysis

Because the primary sampling unit for the study was the school, it was necessary to make adjustments for the clustered survey design in the analyses (using the 'svy' commands in Stata). An equal number of classes were selected in each school regardless of school size. Data were therefore reweighted to adjust for unequal probabilities of selection.

Cross-sectional analysis was carried out on the data collected from pupils in 2001. In univariable analyses, crude odds ratios (ORs) were calculated for the association between each variable and depression using logistic regression. Previous literature has shown that sex is an important predictor of depression [33]. Other potential confounders identified from the univariable analyses fell into three categories: (1) general health, (2) health behaviours and (3) sociodemographic variables. Confounders were assessed using Mantel-Haenszel methods and univariable logistic regression analysis. Effect modification was investigated by looking at the stratum-specific ORs. Body mass index (BMI) was not considered as a confounder because theoretically it may be on the causal pathway [12].

Multivariable analysis was carried out using logistic regression. For the cross-sectional analysis, the continuous physical activity variable was used as the main exposure. Potential confounders were added to the model in groups. If adding a confounder or group of confounders resulted in an improvement in model fit, the confounders were retained in the model.

Longitudinal analysis was restricted to pupils who were surveyed in both 2001 and 2003. Multivariable longitudinal analysis was carried out to examine the impact of a change in physical activity on depression (using the continuous variable of change in physical activity described above). All analyses adjusted for physical activity and depressive symptoms at baseline. Confounders were added to the model in the same way as for the cross-sectional analysis. The Wald test was used to assess goodness-of-fit. Analyses were also carried out to examine direction of causality, looking at whether participants who developed depressive symptoms between baseline and follow-up had higher odds of changing their level of physical activity.

## Results

### Survey

Of the total of 3,322 pupils eligible for survey the overall response was 84% (2,789 pupils). There was some evidence for lower response among pupils who were eligible for school meals (*P *= 0.055) and strong evidence for lower response amongst white pupils (*P *< 0.0001). A total of 2,093 (75%) pupils were also followed-up in 2003. Some groups were less likely to be followed-up: those depressed in 2001 (*P *= 0.002), girls (*P *= 0.005), those eligible for free school meals (*P *= 0.002) and white pupils (*P *≤0.0001).

### The sample

Table 1 describes the sample at baseline. Nearly a quarter of pupils were depressed. Levels of physical activity varied, with most pupils undertaking between 0.5 to 2-3 h of physical activity a week.

**Table 1:** Characteristics of the sample at baseline.

	N	Percentage adjusted for survey design
Depressive symptoms:		
Case on SMFQ	671	24.5
Not a case on SMFQ	2,054	75.5
Physical activity:		
None	317	11.5
About 0.5 h	787	28.6
About 1 h	623	22.7
About 2-3 h	609	22.3
About 4-6 h	239	8.7
7 h or more	171	6.2
Sex:		
Male	1,356	48.8
Female	1,433	51.1
Year group:		
Year 7	1,381	49
Year 9	1,408	51
Ethnicity:		
White UK	581	20.8
White other	161	5.9
Bangladeshi	690	25.6
Indian	250	9
Pakistani	184	6.8
Black Caribbean	166	6
Black African	279	10.1
Black British	121	4.3
Mixed ethnicity	193	7
Other	124	4.5
Religious group:		
None	325	11.5
Jewish	3	0.1
Christian	994	35.7
Muslim	1,169	42.7
Hindu	101	3.7
Sikh	72	2.7
Agnostic/atheist/don't know	59	2.1
Other	43	1.6
Either parent employed:		
Neither	1,004	37.5
At least one	1,681	62.5
Eligibility for free school meals:		
Not eligible	1,338	51.9
Eligible	1,217	48.1

SMFQ = Short Moods and Feelings Questionnaire.

### Cross-sectional univariable analysis

Table 2 shows the univariable analysis. There was strong evidence that physical activity was inversely associated with depressive symptoms (*P *= 0.003). It was found that 1 h more physical activity per week was associated with an 11% decrease in the odds of depressive symptoms (OR = 0.89, 95% CI 0.85 to 0.93).

**Table 2:** Odds of depressive symptoms in 2001 by health and health behaviours (results of univariable logistic regression analysis).

	OR	95% CI	*P *value for model	N
Physical activity, h/week:				
None	1		0.003	2,705
About 0.5 h	0.94	0.71 to 1.25		
About 1 h	0.9	0.67 to 1.21		
About 2-3 h	0.67	0.49 to 0.91		
About 4-6 h	0.58	0.37 to 0.90		
More than 7 h	0.48	0.30 to 0.77		
Physical activity, h/week (continuous variable)	0.89	0.85 to 0.93	<0.0001	2,705
General health:				
Very good	1		<0.0001	2,717
Good	1.15	0.90 to 1.47		
Fair	1.65	1.36 to 2.00		
Bad	3.81	2.17 to 6.67		
Very bad	5.23	1.82 to 15.00		
Presence of long-standing illness/disability:				
No	1		0.003	2,648
Yes	1.4	1.13 to 1.73		
Body mass index:				
Below 85th percentile	1		0.006	2,462
85th-95th percentile (overweight)	1.04	0.72 to 1.49		
Over 95th percentile (obese)	1.45	1.17 to 1.81		
Cigarette smoking:				
Never smoked	1		0.021	2,704
Less than 1 a week	1.39	1.05 to 1.84		
1 or more a week	1.84	1.13 to 2.98		
Units alcohol drunk last week:				
Did not drink last week	1		0.037	2,679
1-5.5 units	1.06	0.59 to 1.89		
More than 6 units	3.18	1.32 to 7.68		
Ever tried drugs:				
No	1		0.001	2,719
Yes	1.49	1.21 to 1.84		
Daily portions fruit and vegetables:				
0-2	1		0.045	2,588
3-4	0.91	0.72 to 1.15		
5 or more	1.15	0.89 to 1.48		

OR = odds ratio.

With the exception of sex, for which there was very strong evidence for an association with depressive symptoms (*P *< 0.0001), there was no evidence that the sociodemographic variables were associated with depressive symptoms (data not reported). Girls had nearly twice the odds of depressive symptoms of boys (OR = 1.82, 95% CI 1.49 to 2.24).

### Multivariable analysis: baseline

Evidence for effect modification was found for sex (*P *= 0.047). Models are presented separately for boys and girls. Potential confounders were identified in two categories: (1) general health and (2) health behaviours. Variables measuring health were self-reported general health and presence of a long-standing illness or disability. Health behaviours included cigarette smoking, units of alcohol drunk in a week, whether the pupil had tried drugs and daily portions of fruit and vegetables consumed. Table 3 shows the multivariable regression analyses stratified by sex.

**Table 3:** Odds of depressive symptoms in 2001 per hour of physical activity (results of multivariable logistic regression analysis).

	Boys, N = 1,195			Girls, N = 1,269		
	OR	95% CI (*P *value)	Wald *P *value^a^	OR	95% CI (*P *value)	Wald *P *value
Crude odds	0.92	0.87 to 0.97 (0.003)		0.94	0.88 to 1.01 (0.087)	
Adjusted for general health/long-standing illness	0.92	0.87 to 0.98 (0.011)	0.0001	0.95	0.89 to 1.02 (0.180)	0.0004
Adjusted for general health/long-standing illness and health behaviours	0.92	0.85 to 0.99 (0.023)	0.0275	0.92	0.85 to 1.00 (0.044)	0.0003

^a^Test for improvement in model fit.

OR = odds ratio.

There was strong evidence for an association between physical activity and depressive symptoms for boys (*P *= 0.003). Each additional hour of physical activity per week was associated with a reduction in odds of depressive symptoms of about 8% (OR = 0.92, 95% CI 0.87 to 0.97). Adjusting for confounders resulted in a little change in the odds of depressive symptoms in the final model (OR = 0.92, 95% CI 0.85 to 0.99) but evidence for an association between depressive symptoms and physical activity remained (*P *= 0.023). After adjustment for confounders, there was also some evidence for an association between higher physical activity and reduced odds of depressive symptoms for girls (OR = 0.92, 95% CI 0.85 to 1.00; *P *= 0.044).

### Multivariable analysis: baseline and follow-up

Table 4 shows results of multivariable analysis for the association between a change in physical activity level between baseline and follow-up and depressive symptoms at follow-up, stratified by sex.

**Table 4:** Odds of depressive symptoms in 2003 per hour change in level of physical activity between 2001 and 2003 as main explanatory variable^a^.

	Boys, N = 812			Girls, N = 863		
	
	OR	95% CI (*P *value)	Wald *P *value	OR	95% CI (*P *value)	Wald *P *value
Crude odds	0.98	0.89 to 1.09 (0.715)		0.95	0.87 to 1.03 (0.179)	
Adjusted for general health/long-standing illness	0.99	0.89 to 1.09 (0.810)	0.4303	0.95	0.88 to 1.03 (0.226)	0.5667
Adjusted for health behaviours	0.99	0.89 to 1.10 (0.873)	0.6241	0.95	0.87 to 1.04 (0.284)	0.0638

^a^All analyses adjusted for baseline depressive symptoms and physical activity.

OR = odds ratio.

The longitudinal analysis indicated no significant association between a change in level of physical activity and odds of depressive symptoms at follow-up, although the direction of effect was the same as found in the cross-sectional analyses. There was no evidence for reverse causality; participants who became depressed between baseline and follow-up did not have higher odds of changing their physical activity levels.

## Discussion

### Principal findings

In a cross-sectional study we found evidence for an association between physical activity and depressive symptoms in adolescents; an increase in physical activity of about 1 h a week was associated with an 8% decrease in the odds of depressive symptoms in both boys and girls. There was no statistically significant association between physical activity and depressive symptoms in a longitudinal analysis, although the direction of effect was the same.

### Strengths and weaknesses

This study is novel in that no previous study on adolescents has examined the question of direction of causality. Although it is impossible to prove a causal association with observational data, the Bradford Hill criteria provide a framework for making an overall judgement about whether a causal association is likely [34]. One important criterion is temporal sequence of association. The design of RELACHS provided an ideal opportunity to look at temporal sequence of association and found no evidence for reverse causality. A further criterion for causality outlined by Bradford Hill was evidence of a dose response relationship. The univariable analysis for the full categorical variable suggests that a dose response relationship exists, although some caution must be exercised as the confidence intervals overlap.

The composition of the sample was broadly similar to the school population for the three boroughs in terms of ethnicity and eligibility for free school meals. It is unclear how far the results would be generalisable to adolescents outside East London. A response rate of 84% is reasonable and a number of strategies were used to encourage reliable response for an adolescent sample. The overall follow-up was 75% and there was no difference in follow-up by level of physical activity. However, certain groups were less likely to be followed-up. These were: adolescents who were depressed at baseline, girls, those eligible for free school meals and white pupils. This is most likely to be due to these groups being absent from school on the day that the questionnaire was administered or because they had left the school between baseline and follow-up. Patterns of follow-up may have had an impact on the associations found. Those eligible for free school meals were less likely to undertake regular physical activity. Girls were more likely to have depression and were less likely to undertake regular physical activity; this may have contributed to an underestimation of the strength of association between physical activity and depression at follow-up.

All of the measures, with the exception of height and weight, were self-reported and there are no objective measures with which to compare the pupils' answers. However, for the main outcome, the SMFQ is a well accepted instrument. It has been found to be a reliable measure of a core depression construct in adolescents to be used in epidemiological studies [35]. Regarding the main exposure variable, it has been questioned by some commentators whether self-report data is an adequate measure of physical activity. It has been suggested that self-reported physical activity is only weakly correlated with actual energy expenditure [36,37]. Pupils with depressive symptoms may be less likely to recall periods of physical activity than those that are not depressed due to a tendency to underestimate their level of activity/achievement. As students get older, it may be less fashionable to be active; physical activity at follow-up may therefore be underestimated. This may partly account for the failure to find a longitudinal association between physical activity and depressive symptoms. Another important limitation in relation to the physical activity variable is that it measures physical activity outside school; activity taken within the school, during physical education classes and break times is not taken into account. Although physical activity classes taken within the school are probably broadly equal across all participants, intensity may have varied considerably amongst pupils. Some pupils are also likely to be more active than others during break times.

The study may suffer from a lack of power, particularly in the longitudinal analysis. An association between physical activity and depression was found cross-sectionally for boys and girls. A lack of evidence for an association in the longitudinal analysis does not mean that there is not a true association. As discussed above, the self-report measure may not adequately reflect actual energy expenditure. The way in which the variable for change in physical activity was created was approximate and leaves room for error. The combination of these weaknesses may have led to a lack of power to detect the potential longitudinal association between depressive symptoms and physical activity.

A weakness of previous studies has been a failure to control for a number of potentially important confounders including chronic illness and general health measures. This study was able to adjust for both as well as for health behaviours. However, there is still a possibility of residual confounding.

### Previous work

The results of the cross-sectional analysis are in line with previous work in finding an association between depressive symptoms and physical activity [12-20]. Few studies have stratified the analysis by gender. Allison *et al*. found no evidence for a gender interaction, contrary to their expectations. However, their response rate was low and they do not provide information on the degree of missing data [13]. Brown *et al*. looked at the odds of suicidal behaviour separately for boys and girls and found a stronger association for boys than for girls [38]. These results closely mirror those reported here.

Only three longitudinal studies have been undertaken to examine the association between physical activity and depression. A Norwegian study found an association between hours of physical activity a week at age 15-16 and depression 3 years later for boys but not for girls [10]. A longitudinal study in Germany found that subjects with regular physical activity at baseline had a lower overall incidence of mental disorder [22]. They found no association between physical activity and major depression. Their findings may be due to a lack of power once the analysis was broken down by specific disorder. Motl *et al*.'s study in the US found an overall association between a change in the frequency of physical activity and change in depression [21]. No longitudinal association was found in the analyses reported here. However, this may be due to lack of power; the direction of effect is the same as in the cross-sectional analyses.

### Recommendations for further research

Further research is needed to establish causality. The most direct test would be an experimental design. However, a problem with these studies is that they use selected samples. Two groups are always underrepresented: those who already exercise heavily and those who are persistently sedentary and do not participate, or drop out. Working within the framework of cohort studies may therefore be the best strategy. A key priority is to be able to look at the relationship between physical activity and depressive symptoms within a cohort with sufficient numbers of participants to detect potential associations. The study reported here was limited by small numbers once the sample was stratified by sex. One way to proceed would be to press for items on physical activity to be included consistently in large longitudinal studies such as the Millennium Cohort Study; the children in this sample are currently 10 years old. Efforts are needed to develop robust items for recording physical activity, with objective as well as self-report measures if possible. Regarding the main outcome, the SMFQ is a well accepted self-report instrument for measuring the presence of depressive symptoms in adolescents [35]. However, future research might consider using a measure involving a structured interview.

Further research is needed into the mechanisms linking physical activity and depressive symptoms. The findings suggest that the association cannot be explained by social class differences within this population; it would therefore be interesting to explore whether any of the major psychosocial hypotheses for the link are supported. The absence of a firm mechanism linking physical activity and depression may be a factor in explaining why physical activity has not been more universally taken up as a means of preventing or treating depression in the UK, although in some countries (for example, Belgium) it has been accepted and established in delivery systems [5]. There is an urgent need to collaborate with medical and neurobiological scientists to explore these mechanisms.

## Conclusions

This study suggests that there is an association between physical activity and depression in adolescents. There has been limited work looking at the relationship between physical activity and depression in adolescents, particularly longitudinally. The findings are tentative; before recommendations can be made to promote physical activity specifically in prevention or treatment, more research is required.

## Competing interests

The authors declare that they have no competing interests.

## Authors' contributions

CR had the idea for the paper, planned and carried out the data analysis and wrote up the results. PE advised on the data analysis and reviewed and edited the paper. KB planned the study and reviewed the manuscript. RMV planned the study and reviewed the manuscript. ST planned the study and reviewed the manuscript. SAS planned and executed the study, reviewed the manuscript and is guarantor.

## Pre-publication history

The pre-publication history for this paper can be accessed here:

http://www.biomedcentral.com/1741-7015/8/32/prepub
